# The Clinical Frailty Scale (CFS) as an Independent Prognostic Factor for Patients ≥80 Years with Small Bowel Obstruction (SBO)

**DOI:** 10.1007/s11605-023-05820-8

**Published:** 2023-09-06

**Authors:** Vito Laterza, Marcello Covino, Carlo Alberto Schena, Andrea Russo, Sara Salini, Davide Della Polla, Nicola de’Angelis, Giuseppe Quero, Vincenzo Tondolo, Antonio La Greca, Giuseppe Merra, Gabriele Sganga, Antonio Gasbarrini, Francesco Franceschi, Francesco Landi, Sergio Alfieri, Fausto Rosa

**Affiliations:** 1 Digestive Surgery, Fondazione Policlinico Universitario A. Gemelli, IRCCS, Largo A. Gemelli, 8,, 00168 Rome, Italy; 2 Università Cattolica del Sacro Cuore, Rome, Italy; 3 Emergency Medicine, Fondazione Policlinico Universitario A. Gemelli, IRCCS, Rome, Italy; 4 Geriatrics Department, Fondazione Policlinico Universitario A. Gemelli, IRCCS, Rome, Italy; 5 Emergency and Trauma Surgery, Fondazione Policlinico Universitario A. Gemelli, IRCCS, Rome, Italy; 6 Unit of Colorectal and Digestive Surgery, DIGEST Department, Beaujon University Hospital, AP-HP, University of Paris Cité, Clichy, Paris, France; 7 Section of Clinical Nutrition and Nutrigenomic, Department of Biomedicine and Prevention, University of Rome Tor Vergata, Rome, Italy; 8 Internal Medicine and Gastroenterology, Fondazione Policlinico Universitario A. Gemelli, IRCCS, Rome, Italy

**Keywords:** Small bowel obstruction (SBO), Frailty, Elderly, Emergency surgery, Non-operative management (NOM)

## Abstract

**Background:**

SBO is a potentially life-threatening condition that often affects older patients. Frailty, more than age, is expected to play a crucial role in predicting SBO prognosis in this population. This study aims to define the influence of Clinical Frailty Scale (CFS) on mortality and major complications in patients ≥80 years with diagnosis of SBO at the emergency department (ED).

**Methods:**

All patients aged ≥80 years admitted to our ED for SBO from January 2015 to September 2020 were enrolled. Frailty was assessed through the CFS, and then analyzed both as a continuous and a dichotomous variable. The endpoints were in-hospital mortality and major complications.

**Results:**

A total of 424 patients were enrolled. Higher mortality (20.8% vs 8.6%, *p*<0.001), longer hospital stay (9 [range 5–14] days vs 7 [range 4–12] days, *p*=0.014), and higher rate of major complications (29.9% vs 17.9%, *p*=0.004) were associated with CFS ≥7. CFS score and bloodstream infection were the only independent prognostic factors for mortality (OR 1.72 [CI: 1.29–2.29], *p*<0.001; OR 4.69 [CI: 1.74–12.6], *p*=0.002, respectively). Furthermore, CFS score, male sex and surgery were predictive factors for major complications (OR 1.41 [CI: 1.13–1.75], *p*=0.002; OR 1.67 [CI: 1.03–2.71], *p*=0.038); OR 1.91 [CI: 1.17–3.12], *p*=0.01; respectively). At multivariate analysis, for every 1-point increase in CFS score, the odds of mortality and the odds of major complications increased 1.72-fold and 1.41-fold, respectively.

**Conclusion:**

The increase in CFS is directly associated with an increased risk of mortality and major complications. The presence of severe frailty could effectively predict an increased risk of in-hospital death regardless of the treatment administered. The employment of CFS in elderly patients could help the identification of the need for closer monitoring and proper goals of care.

## Introduction

Small bowel obstruction (SBO) accounts for 15% of emergency department (ED) admissions for abdominal pain and its burden is estimated to be higher in patients older than 80 years [1–3]. Adhesions, hernias, and neoplasms are the leading causes of SBO in nine out of ten patients [4]. Therefore, the occurrence of SBO increases proportionally with the age of patients requiring ED admission [4]. Furthermore, despite a similar clinical presentation, the observed mortality is much higher in octogenarians than in younger patients, due to the remarkable rate of cardiovascular and metabolic comorbidities [5].

The burden of SBO in older patients is even more relevant considering that, according to the World Health Organization (WHO), the segment of the population older than 80 years is expected to triple in the next decades, reaching 426 million people worldwide by 2050 [6].

Nevertheless, geriatric patients represent a heterogeneous population in terms of physical and neurological performances, comorbidities, and resilience to acute insults [7, 8]. Consequently, prognosis and mortality after SBO may vary widely [7, 8].

To overcome the mismatch between chronological age, comorbidities, and older patients’ general health status and prognosis, the concept of frailty was proposed [9, 10]. In particular, the Clinical Frailty Scale (CFS) is a viable and reproducible tool for assessing frailty [11], and its reliability as an independent predictor of mortality has already been validated for elective and emergency surgical and nonsurgical populations [12–17]. However, its effectiveness for SBO in octogenarians remains unproven in the emergency setting.

This study aims to define the influence of frailty through CFS on mortality and major complications in patients ≥ 80 years or older with a proven diagnosis of SBO at the ED.

## Methods

### Study Design

This is a single-centre, prospective, observational cohort study, performed in the ED of a tertiary care University Hospital (Fondazione Policlinico Universitario “Agostino Gemelli” IRCCS of Rome) with an average attendance of about 75,000 patients per year (more than 87% adults).

### Inclusion and Exclusion Criteria

After the approval of the Institutional Review Board (Fondazione Policlinico Universitario Agostino Gemelli IRCCS, Rome, Italy, ID: 5121/2022), all patients aged ≥80 years consecutively admitted to our ED for SBO from January 2015 to September 2020 were enrolled, regardless of operative or non-operative management (NOM).

The denial to participate in the study and the lack of a complete frailty assessment represented exclusion criteria.

The clinical records of the eligible patients were retrospectively collected from a prospectively maintained databases and identified using the International Classification of Disease, 9th Revision, Clinical Modification (ICD-9-CM) codes [18], as follows: 560.0, 560.80, 560.81, 560.89, 560.90.

### Study Variables

The following demographic and clinical data were collected: age and gender; frailty assessed via CFS as reported by Rockwood et al. [11], clinical presentation at admission (abdominal pain, fever, vomit, gastrointestinal bleeding), vital signs (heart rate, blood pressure, peripheral oxygen saturation, body temperature) and laboratory results (haemoglobin, white blood cells count, serum glucose and creatinine, prothrombin time test, fibrinogen), clinical history and comorbidities (coronary artery disease, chronic heart failure, cerebrovascular disease, dementia, peripheral artery disease, connective tissue disease, cirrhosis, diabetes, chronic obstructive pulmonary disease, chronic kidney disease, malignancy), including Charlson Comorbidity Index (CCI) [19], aetiology (malignancies, surgical adhesions, hernias, volvulus), 30-day mortality, length of hospital stay (LOS), calculated from the time of ED admission to discharge or death, major complications, defined as the need for prolonged stay into intensive care unit (> 96 h), the occurrence of sepsis (defined according to sepsis-3 criteria [20]) or peritonitis, and death.

### Small Bowel Obstruction Assessment

SBO was diagnosed after ED admission through clinical examination, laboratory tests, and imaging. Specifically, SBO diagnosis was radiologically confirmed by an abdominal CT scan in all patients. NOM, surgical management, and their timing were previously described [21]. With ‘interventional procedure’, any interventional endoscopic or radiological procedure with drainage insertion was considered (i.e. bowel decompression, fluid evacuation).

### Clinical Frailty Scale Assessment

**Fig. 1:**
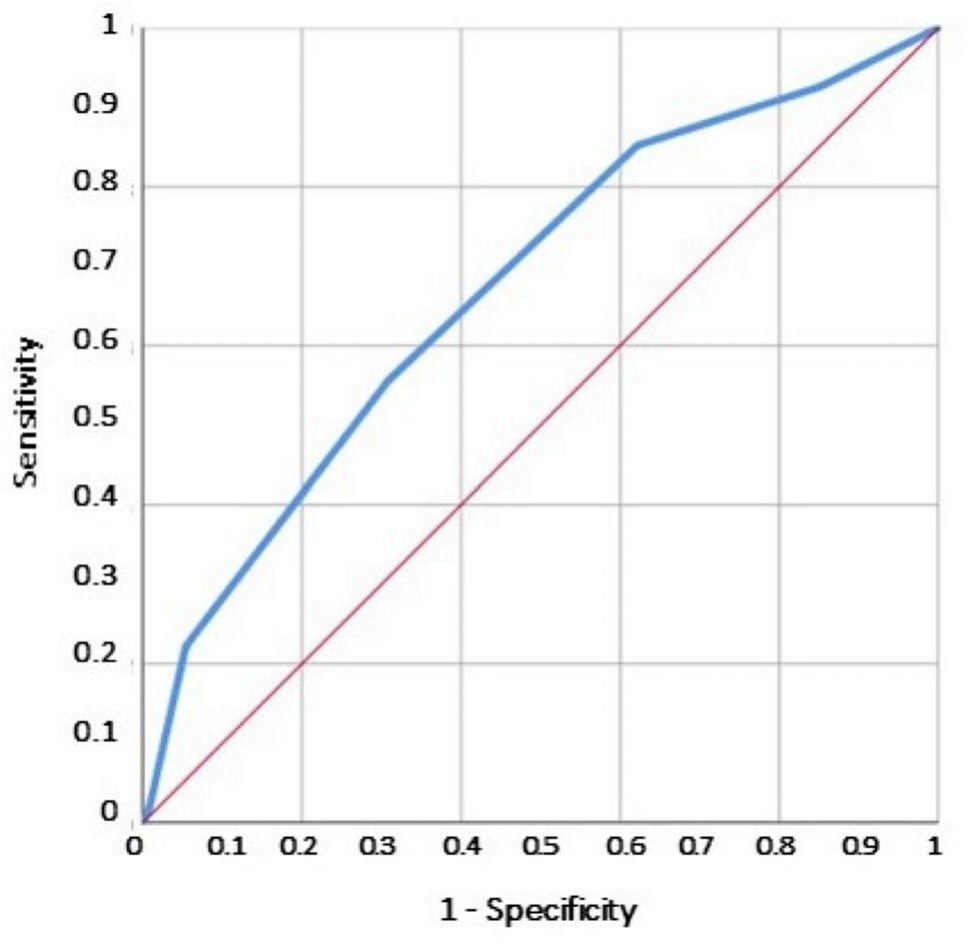
Receiver operating characteristics analysis (ROC) of the Clinical Frailty Score (CFS) for the prediction of all-cause in-hospital death. According to the Youden index J, the best discriminating value was ≥ 7 which corresponds to severe frailty

Frailty was assessed by the CFS [11]. CFS was analyzed both as a continuous and a dichotomous variable. In the latter case, patients were divided into two groups according to CFS: mild or moderately frail for CFS ≤6, and severely frail in case of CFS ≥ 7. The obtained CFS score was evaluated for overall accuracy in identifying patients at risk of frailty by receiver operating characteristic (ROC) curve analysis. The sensitivity and specificity were identified for each score level by ROC analysis. The optimal dividing cut-off associated with CFS score was obtained by Youden's index, and a two-sided *p*-value ≤ 0.05 was regarded as significant. The best discriminating value was ≥ 7 which corresponds to severe frailty (Fig. 1).

### Study Endpoints

The endpoints of this study were the occurrence of in-hospital death and the cumulative occurrence of major complications (defined as the occurrence of any among septic shock, need for ICU admission, and death).

### Statistical Analysis

Continuous variables were reported as median (interquartile range) and compared by the Mann–Whitney U test. Categorical variables were reported as absolute numbers (percentage) and statistically compared by the chi-square test (with Fisher test if appropriate). Multiple comparisons were assessed for false discovery rate (FDR) by the Benjamini–Hocheberg method.

Receiver operating characteristic (ROC) curve analysis was performed to test the specificity and sensitivity of the CFS predicting endpoints (in-hospital mortality and major complications). ROC Youden’s index J was used to determine the best cut-off values for CFS to define outcomes. The c-statistic evaluates CFS discrimination and represents the area under the ROC curve (AUC). A value of 0.5 is equivalent to chance; a value of 1.0 indicates perfect discrimination. Nevertheless, the CFS score was also included in the multivariate analysis as a continuous variable, achieving the odds of increased risk for each point of CFS value.

The variables reaching statistical significance at univariate analysis were considered for the multivariate logistic regression model. Multivariate models excluded the single items composing any derived variable, both to avoid model overfitting and parameter overestimation. The risk of intrahospital death and major complications was expressed as odds ratio (OR) and 95% confidential interval (CI). A 2-sided *p* ≤ 0.05 was considered significant in all the analyses.

Data were analyzed by SPSS v25® (IBM, Armonk, NY, USA).

## Results

### Patients’ Characteristics

During the study period, a total of 424 patients ≥ 80 years (239 females and 185 males with a median age of 85 years) were admitted to our ED with a diagnosis of SBO. The clinical and demographic characteristics of the study population are reported in Table 1. Overall, 280 patients (66%) had a CFS score between 1 and 6, and 144 patients (34%) had a CFS score of 7–9.

**Table 1:** Clinical characteristics of enrolled patients according to the frailty as assessed by the Clinical Frailty Scale (CFS) at the emergency department admission

	All cases N 424	CFS 1-6 N 280	CFS 7-9 N 144	*P* value
Age	85 [82–89]	84 [81–87]	88 [84–91]	<0.001
Female	239 (56.4%)	167 (59.6%)	72 (50%)	0.037
*ED presentation*				
Fever	361 (85.1%)	240 (85.7%)	121 (84%)	0.37
Abdominal pain	281 (66.3%)	201 (71.8%)	80 (55.6%)	0.001
Vomit	209 (49.3%)	145(51.8%)	64 (44.4%)	0.092
Gastrointestinal bleeding	22 (5.2%)	16 (5.7%)	6 (4.2%)	0.33
*Treatment*				
Medical treatment	246 (58.1%)	157 (56.1%)	89 (61.8%)	
Percutaneous procedures	18 (4.2%)	10 (3.6%)	8 (5.6%)	0.17
Ostomy creation	56 (13.2%)	31 (11.1%)	47 (32.6%)	0.05
Surgery (overall)	160 (37.7%)	113 (40.3%)	47 (32.6%)	0.073
*Coexistent acute infections*				
Bloodstream infection	23 (5.4%)	17 (6.1%)	6 (4.2%)	0.28
Any infections	73 (17.2%)	40 (14.3%)	33 (22.9%)	0.019
*Comorbidities*				
CCI	6 [4–8]	6 [4–7]	7 [5–7]	<0.001
CFS	6 [5–7]	5 [5–6]	7 [7–8]	<0.001
History of CAD	60 (14.2%)	40 (14.3%)	20 (13.9%)	0.52
CHF	45 (10.6%)	23 (8.2%)	22 (15.3%)	0.021
Cerebrovascular disease	32 (7.5%)	19 (6.8%)	13 (9.0%)	0.26
Dementia	50 (11.8%)	16 (5.7%)	34 (23.6%)	<0.001
COPD	58 (13.7%)	37 (13.2%)	21 (14.6%)	0.401
Chronic Kidney disease	67 (15.8%)	35 (12.5%)	32 (22.2%)	0.008
Diabetes	48 (11.3%)	33 (11.8%)	15 (10.4%)	0.673
Oncological disease	166 (39.2%)	106 (37.9%)	60 (41.7%)	0.25
Metastatic Cancer	64 (15.1%)	28 (10%)	36 (25%)	<0.001
*Aetiology*				
Malignancy	120 (28.3%)	71 (25.4%)	49 (34%)	0.04
Surgical adhesions	61 (14.4%)	50 (17.9%)	11 (7.6%)	0.003
Hernia	35 (8.3%)	28 (10%)	7 (4.9%)	0.047
Volvulus	19 (4.5%)	10 (3.6%)	9 (6.3%)	0.155
Perforation	16 (3.8%)	11 (3.9%)	5 (3.5%)	0.525
*Outcomes*				
Death (all causes)	54 (12.7%)	24 (8.6%)	30 (20.8%)	<0.001
Length of stay (LOS)	8 [5–13]	7 [4–12]	9 [5–14]	0.014
Major complications	84 (19.8%)	38 (13.6%)	46 (31.9%)	<0.001

*Abbreviations*: *CCI*, *Charlson comorbidity index*; *CFS*, *Clinical Frailty Scale*; *CAD*, *coronary artery disease*; *CHF*, *chronic heart failure*; *COPD*, *chronic obstructive pulmonary disease*

The most common clinical signs at ED admission were fever (85.1%) and abdominal pain (66.3%), but only the latter was significantly more frequent in the group including non-to-moderately frail patients (*p*=0.001). Moreover, patients with severe frailty (CFS ≥7) were characterized by higher median CCI (7 [range 5–7] vs 6 [range 4–7], *p*<0.001).

Malignancies represented the most frequent aetiology in the severely frail group (34% vs 25.4%, *p*=0.04) while a higher incidence of surgical adhesions and hernias was found in mild-to-moderately frail patients (17.9% vs 7.6%, *p*=0.003; and 10% vs 4.9%, *p*=0.047, respectively).

Comparing outcomes, higher mortality (20.8% vs 8.6%, *p*<0.001), longer LOS (9 [range 5–14] days vs 7 [range 4–12] days, *p*=0.014), and a higher rate of major complications (29.9% vs 17.9%, *p*=0.004) were significantly associated with higher frailty index (CFS ≥7). At the same time, an ostomy was needed significantly more frequently in patients with severe frailty (*p*=0.05).

### Factors Associated with Mortality

The association of the study variables with mortality is shown in Table 2.

**Table 2:** Factors associated with mortality

	Survived *N* 370	Deceased *N* 54	Univariate *p* value	Odds Ratio [95% interval]	Multiv. *p* value
CFS 1–6	256 (69.2%)	24 (44.4%)			
CFS 7–9	114 (30.8%)	30 (55.6%)	<0.001		
CFS	6 [5–7]	7 [6–7]	<0.001	1.72 [1.29–2.29]	<0.001
Age	85 [82–89]	85 [81–91]	0.468		
Sex (male)	153 (41.4%)	32 (59.3%)	0.013	1.73 [0.93–3.23]	0.084
*ED presentation*					
Fever	55 (14.9%)	8 (14.8%)	0.59		
Abdominal pain	249 (67.3%)	32 (59.3%)	0.15		
Vomit	185 (50%)	24 (44.4%)	0.27		
Gastrointestinal bleeding	17 (4.6%)	5 (9.3%)	0.134		
Perforation	11 (3%)	5 (9.3%)	0.04	2.37 [0.71–7.89]	0.158
*Treatment*					
Medical treatment	213 (57.6%)	33 (61.1%)			
Percutaneous procedures	15 (4.1%)	3 (5.6%)	0.408		
Ostomy creation	50 (13.5%)	6 (11.1%)			
Surgery (overall)	142 (38.4%)	18 (33.3%)	0.289		
*Coexistent acute infections*					
Bloodstream infection	14 (3.8%)	9 (16.7%)	0.001	4.69 [1.74–12.6]	0.002
Any infections	62 (16.8%)	11 (20.4%)	0.31		
*Comobidities*					
CCI	6 [4–8]	6 [5–8]	0.273		
CFS	6 [5–7]	7 [6–7]	<0.001		
History of CAD	51 (13.8%)	9 (16.7%)	0.35		
CHF	38 (10.3%)	7 (13%)	0.34		
Cerebrovascular disease	27 (7.3%)	5 (9.3%)	0.39		
Dementia	41 (11.1%)	9 (16.7%)	0.166		
COPD	50 (13.5%)	8 (14.8%)	0.465		
Chronic kidney disease	59 (15.9%)	8 (14.8%)	0.509		
Oncological disease	144 (38.9%)	22 (40.7%)	0.454		
Metastatic cancer	55 (14.9%)	9 (16.7%)	0.43		
*Aetiology*					
Malignancy	100 (27%)	20 (37%)	0.088		
Non malignant	102 (27.6%)	3 (5.6%)	<0.001	0.21 [0.06–0.71]	0.012
(i) Adhesions	60 (16.2%)	1 (1.9%)	0.001		
(ii) Hernia	34 (9.2%)	1 (1.9%)	0.045		
(iii) Volvulus	18 (4.9%)	1 (1.9%)	0.027		

*Abbreviations*: *CCI*, *Charlson comorbidity index*; *CFS*, *Clinical Frailty Scale*; *CAD*, *coronary artery disease*; *CHF*, *chronic heart failure*; *COPD*, *chronic obstructive pulmonary disease*

At univariate analysis, CFS score (*p*<0.001), CFS ≥7 (*p*<0.001), male sex (*p*=0.013), intestinal perforation (*p*=0.04), and bloodstream infections (*p*=0.001) were significantly associated with higher mortality. Conversely, as far as aetiology is concerned, surgical adhesions (*p*=0.001), hernia (*p*=0.045), and volvulus (*p*=0.027) were associated with a lower mortality rate, whereas malignancy (*p*=0.088) was associated with a higher death rate.

After the adjustment for covariates, only CFS score (OR 1.72 [CI: 1.29–2.29], *p*<0.001) and blood stream infection (OR 4.69 [CI: 1.74–12.6], *p*=0.002) emerged as independent risk factors for mortality, whereas non malignant aetiology (OR 0.21 [CI: 0.06–0.71], *p*=0.012) resulted as protective factor at multivariate analysis. For every 1-point increase in CFS score, the odds of mortality increased 1.72-fold.

### Factors Associated with the Occurrence of Major Complications

The association of the study variables with complications is shown in Table 3.

**Table 3:** Factors associated with cumulative major complications (death, sepsis, admission to ICU)

	NOT major complications *N* 340	Major complications *N* 84	Univariate *p* value	Odds ratio [95% interval]	Multiv. *p* value
CFS 1–6	242 (71.2%)	38 (45.2%)			
CFS 7–9	98 (28.8%)	46 (54.8%)			
CFS value	6 [5–7]	6 [6–7]	0.002	1.41 [1.13–1.75]	0.002
Age	85 [82–89]	85 [81–91]	0.485		
Sex (Male)	134 (40.5%)	51 (54.8%)	0.014	1.67 [1.03–2.71]	0.038
*ED presentation*					
Fever	47 (13.8%)	16 (19.0%)	0.228		
Abdominal pain	235 (69.1%)	46 (54.8%)	0.013	0.69 [0.42–1.13]	0.141
Vomit	171 (50.3%)	38 (45.2%)	0.407		
Gastrointestinal bleeding	17 (5.0%)	5 (6.0%)	0.725		
Perforation	12 (3.5%)	4 (4.8%)	0.596		
*Treatment*					
Medical treatment	205 (61.9%)	41 (44.1%)	0.471		
Percutaneous procedures	11 (3.3%)	7 (7.5%)	0.005		
Ostomy	39 (11.5%)	17 (20.2%)	0.034		
Surgery (overall)	124 (36.5%)	36 (42.9%)	0.012	1.91 [1.17–3.12]	0.010
*Comorbidities*					
CCI	6 [4–8]	7 [5–9]	0.045	0.97 [0.86–1.09]	0.649
History of CAD	43 (12.6%)	17 (20.2%)	0.074		
CHF	33 (9.7%)	12 (14.3%)	0.222		
Cerebrovascular disease	24 (7.1%)	8 (9.5%)	0.440		
Dementia	35 (10.3%)	15 (17.9%)	0.054		
COPD	48 (14.1%)	10 (11.9%)	0.597		
Diabetes	31 (9.1%)	17 (20.2%)	0.004		
Chronic kidney disease	55 (16.2%)	12 (14.3%)	0.671		
Oncological disease	127 (37.4%)	43 (46.4%)	0.127		
Metastatic cancer	47 (13.8%)	17 (20.2%)	0.141		
*Aetiology*					
Malignancy	88 (25.9%)	32 (38.1%)	0.026	1.53 [0.78–2.97]	0.213
Non malignant	90 (27.2%)	15 (16.1%)	0.029		
(i) Surgical Adhesions	56 (16.5%)	5 (6.0%)	0.014		
(ii) Hernia	31 (9.1%)	4 (4.8%)	0.194		
(iii) Volvulus	16 (4.7%)	3 (3.6%)	0.653		

*Abbreviations*: *CCI*, *Charlson comorbidity index*; *CFS*, *Clinical Frailty Scale*; *CAD*, *coronary artery disease*; *CHF*, *chronic heart failure*; *COPD*, *chronic obstructive pulmonary disease*

Higher CFS (*p*=0.022), male sex (*p*=0.014), patients presenting with abdominal pain (*p*=0.013), higher CCI (*p*=0.045), diabetes (*p*=0.004), and those with a malignant aetiology (*p*=0.026) had a higher rate of occurrence of major complications. Conversely, those with surgical adhesions had a lower rate of major complications (*p*=0.014).

Surgical treatment (*p*=0.012), including the need for ostomy creation (*p*=0.034), and percutaneous procedures (*p*=0.005) were significantly associated with major complications at univariate analysis.

After adjusting for covariates, only CFS score (OR 1.41 [CI: 1.13–1.75], *p*=0.002), male sex (OR 1.67 [CI: 1.03–2.71], *p*=0.038), and surgical treatment (OR 1.91 [CI: 1.17–3.12], *p*=0.01) were independently predictive of the risk of major complications at multivariate analysis. More specifically, for every 1-point increase in CFS score, the odds of having major complications increased 1.41-fold.

**Fig. 2:**
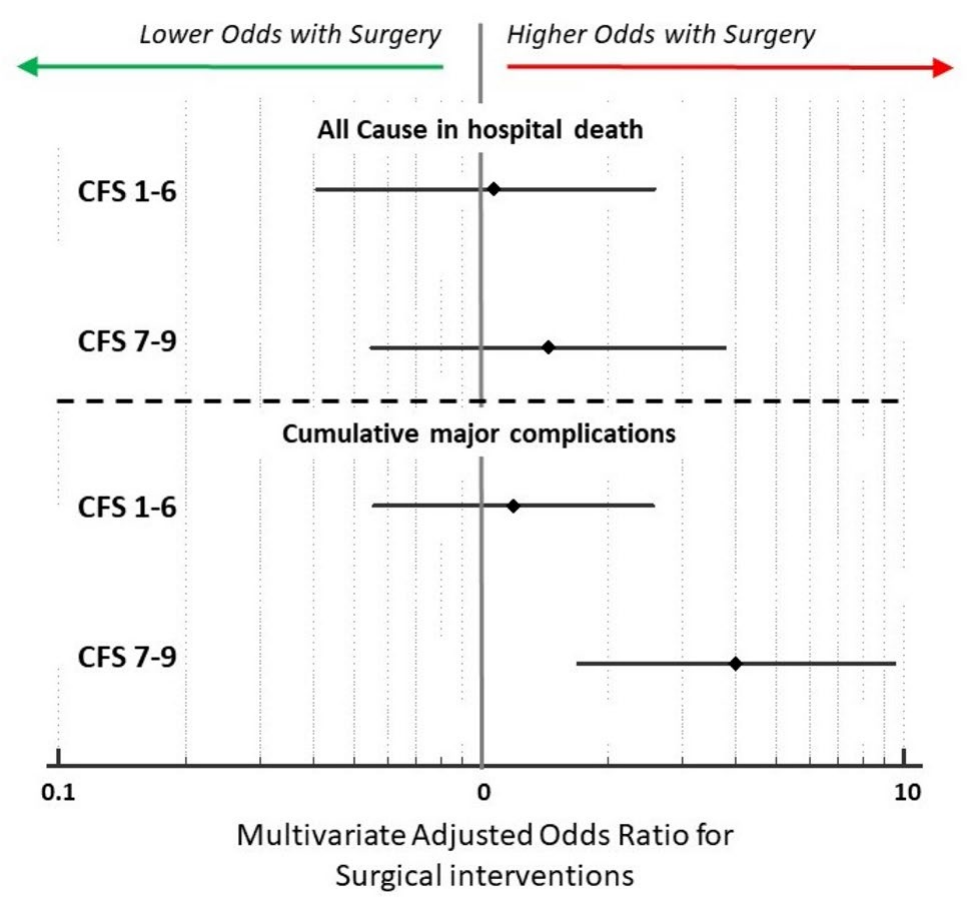
Graphical representation of the adjusted odds for death and major complications calculated for surgical interventions separately in the group of patients with low to moderate frailty, and in the group with severe frailty. Surgery was not associated with in-hospital death in CFS 1-7 and CFS 6-9 groups (OR 1.017 [0.409–2.580] and OR 1.44 [0.549–3.788], respectively). Conversely, surgery was associated with a significant increase in the odds for cumulative major complications in the CFS 7-9 group (*CFS 7-9 group:* OR 4.02 [1.701–9.499]; *CFS 1-6 group:* OR 1.19 [0.553–2.557])

Graphical representation of the adjusted odds for death and major complications were separately calculated for surgical interventions both in the group of patients with low to moderate frailty, and in the group with severe frailty (Fig. 2). Surgery was not associated with in-hospital death in CFS 1-7 and CFS 6-9 groups (OR 1.017 [0.409–2.580] and OR 1.44 [0.549–3.788], respectively).

Conversely, surgical treatment was associated with a significant increase in the odds for cumulative major complications in the CFS 7-9 group (OR 4.02 [1.701–9.499]).

## Discussion

In the present study, we evaluated the outcomes of 424 consecutive octogenarians admitted to the ED with a diagnosis of SBO according to their frailty status, as defined by the CFS. This study represents one of the few attempts in the current literature to evaluate the association between frailty and the outcomes of geriatric patients admitted to the ED with a diagnosis of SBO, and the only experience evaluating these patients through the CFS tool.

SBO is a potentially life-threatening condition that often affects older patients. Due to the progressive ageing of the general population, the incidence of SBO is expected to grow in the next decades, as the occurrence of its leading causes, such as adherence resulting from previous surgery, hernias, and neoplasms, reaches its peak in geriatric patients [1–4, 6].

The management of SBO in this population can be challenging, as a consequence of the higher complexity of elderly patients in terms of comorbidities and capacity to cope with acute stress [5]. Very few studies have shown a higher risk of mortality and complications in octogenarians as compared to younger patients [8, 22–24]. Nevertheless, the geriatric population is widely heterogeneous [7, 8], and stratifying the risk of death and complications after a diagnosis of SBO has become of paramount importance to provide appropriate care and tailored treatment to this group of patients.

As demonstrated by our recent experience [21], the management of SBO in the elderly requires more than just a ‘copy and paste’ of recommendations and guidelines designed for younger patients and, in selected elderly patients with multiple comorbidities or functional impairments, a NOM should always be considered [25, 26], and a comprehensive geriatric assessment is necessary to optimize the diagnostic and clinical strategies [27, 28].

Moreover, as demonstrated by several studies in the current literature, age “as itself” does not represent a comprehensive indicator of the functional reserve of older patients, as it does not provide any reliable information on their comorbidities and general condition [7, 8]. For this reason, the ‘frailty’ concept was introduced as an attempt to encompass the decline in physiologic functions and reduced resilience to internal and external stressors leading to an increased risk of poorer outcomes in the geriatric population [16, 17, 29].

The CFS represent a simple, reproducible, and validated tool for frailty assessment [11, 14], to overcome the complexity of formal evaluations and time-consuming specialized tests, often unavailable in the clinical setting [29–38]. In our study, we found a significantly higher mortality, a longer LOS and a higher rate of complications in the cohort of severely frail patients, regardless of the type of treatment performed. Moreover, CFS was an independent prognostic factor of mortality (*p*<0.001) in the analysed population. Conversely, neither age nor comorbidities were significantly associated to increased mortality in the same patients. On the other hand an increased CFS, male gender and surgery were independently predictive of the risk of major complications. Furthermore, a a point-by-point increase in the CFS score lead to a 1.72-fold and 1.41-fold increase in mortality and major complications, respectively.

These results were not surprising. First of all, both data and common sense suggest increased mortality and morbidity for frail patients undergoing abdominal operations. Secondly, when NOM of SBO is chosen as the first line treatment, it may cause harmful effects on patients’ nutritional status due to the prolonged fasting in a population that is at high risk of malnutrition even before presentation to the emergency room [39, 40], eventually leading to adverse outcomes if the surgical operation is finally performed [39, 40].

Unfortunately, no dedicated recommendations addressing the best management of SBO in frail patients are present in the current international reference guidelines [41].

Our results are in line with those of a recent study by Hwang et al. [42], where the outcomes in terms of mortality and morbidity of 264,670 patients over the age of 65 were investigated. The authors found that frail patients were twice as likely to die as compared to the non-frail population (10% vs 5%, *p*<0.001). Moreover, frailty was found as an independent predictor of in-hospital mortality (aOR 1.82; 95% CI 1.64–2.039), along with other factors such as ethnicity, male gender, increasing age, lower socio-economic status, and undergoing a surgical operation. Nevertheless, the authors employed the Colon Cancer Frailty Index (CCFI) [43] instead of CFS, to stratify their patients into frail vs non-frail ones, making the comparison with our results less obvious.

Therefore, the CFS could reasonably allow the surgeon to better discriminate a surgical procedure or a NOM. Nevertheless, we suggest that this scale must not ignore the physical examination and the case-by-case patient assessment, which remains a fundamental part of the surgeon's evaluation, frequently based on surgeon training and experience.

### Study Limitations

Our study has undoubtedly some limitations. First of all, his retrospective design may have caused potential biases in patients’ inclusion and data analysis. Nevertheless, including not only patients who have undergone surgical procedures but also those who were managed with a NOM may have avoided the active exclusion of frailer patients not eligible for invasive procedures.

Secondly, despite the CFS was analyzed both as a continuous and a dichotomous variable, it should be underlined that considering CFS as a dichotomic variable (the analysis was carried out considering patients with CFS ≤6 and severely frail patients with CFS ≥7 as two distinguished groups) needs validation by future research. Thirdly, although fair, the sample size of the present study was limited, thus limiting the statistical power of our results. Indeed, a sample size of 588 and 786 patients would have been needed in order to reach a statistical power of 0.8 and 0.9, respectively. Finally, this study focused on short-term prognosis and complications, with no evaluation of long-term outcomes.

## Conclusion

In conclusion, our results demonstrated that, in patients ≥80 years with SBO, the presence of severe frailty through a CFS ≥7 could effectively predict an increased risk of death regardless of the treatment administered and a higher risk risk of major complications for patients undergoing surgical procedures. Furthermore, an increasing CFS score is directly associated with an increased risk of mortality and major complications. Therefore, CFS should be considered a useful tool to assess frailty for elderly patients, helping the identification of the need for closer monitoring and the proper goals of care for each patient, avoiding unnecessary and possibly harmful treatments. Other variables, such as the aetiology of SBO, time to operation, and time to enteral nutrition, should be considered to further address the challenges in the management of the growing geriatric population with SBO and improve their outcomes. Future multicenter studies are needed to define dedicated recommendations for octogenarians with SBO to confirm our findings.
